# The effects of action observation training on improving upper limb motor functions in people with stroke: A systematic review and meta-analysis

**DOI:** 10.1371/journal.pone.0221166

**Published:** 2019-08-30

**Authors:** Bingbing Zhang, Laidi Kan, Anqin Dong, Jiaqi Zhang, Zhongfei Bai, Yi Xie, Qianhao Liu, Yuzhong Peng

**Affiliations:** 1 Department of Rehabilitation Medicine, Fifth Affiliated Hospital of Zhengzhou University, Zhengzhou, Henan, China; 2 Department of Biomedical Engineering, The Hong Kong Polytechnic University, Hong Kong, China; 3 Department of Rehabilitation Sciences, The Hong Kong Polytechnic University, Hong Kong, China; 4 Department of Occupational Therapy, Shanghai Yangzhi Rehabilitation Hospital (Shanghai Sunshine Rehabilitation Center), Shanghai, China; 5 Department of Rehabilitation Engineering, Fifth Affiliated Hospital of Zhengzhou University, Zhengzhou, Henan, China; University of Oxford, UNITED KINGDOM

## Abstract

**Background and objective:**

Action observation training (AOT) has been used as a new intervention for improving upper limb motor functions in people with stroke. This systematic review and meta-analysis aims to investigate the effects of AOT on improving upper limb motor functions in people with stroke.

**Methods:**

We searched ten electronic databases to identify randomized controlled trials (RCTs) about the effects of AOT on upper limb motor functions in stroke survivors. Methodological quality of included studies was assessed by the Risk of Bias Tool in the Cochrane Handbook for Systematic Reviews of Interventions. A random-effects meta-analysis was performed by pooling the standardized mean difference (SMD) of upper limb motor outcomes.

**Results:**

Seven studies of 276 participants with stroke were included. Meta-analysis showed a significant effect favoring AOT on improving upper limb motor functions in patients with stroke [SMD = 0.35, 95% confidence interval [CI], 0.10 to 0.61, I^2^ = 10.14%, *p* = 0.007].

**Conclusions:**

AOT appears to be an effective intervention for improving the upper limb motor functions in people after stroke. Further studies need to investigate the neural mechanism underlying the effects of AOT.

## Introduction

Stroke is one of the leading causes of adult disability [1]. Up to 80% of stroke survivors experience upper limb hemiplegia after stroke, and the motor dysfunction could maintain months to years among more than 50% of survivors [2]. The deficit of voluntary upper limb movement was a main factor limiting the life independence in patients after stroke [3]. Restoring the upper extremity functions is one of the major goals in stroke rehabilitation. Numerous upper limb motor rehabilitative strategies have been employed to induce positive neuroplasticity in patients with stroke, such as constraint-induced movement therapy [4], virtual reality-based training [5] and task-oriented training [6]. Studies have demonstrated that cortical reorganization of the lesioned cortical motor system could be induced by these rehabilitative trainings [7, 8]. However, these trainings rely on active movement execution, which is unavailable for those stroke survivors with severe upper limb motor deficits.

Cortical motor system could be activated by action observation (AO) even in the absence of action execution [9]. The premotor cortex and inferior parietal lobe can be activated not only when people are executing an action but also when observing the action performed by others, which is attributed to the assumed human mirror neuron system (MNS) [9]. It is believed that the MNS has functional connections with the dorsal premotor cortex and primary motor cortex [10]. The downstream modulation of MNS on cortical motor system may contribute to the upper limb motor recovery after stroke [11, 12]. Studies in healthy individuals indicated that AO could facilitate the motor skills learning and enhance the corticospinal excitability even in the absence of real motor practice [13, 14]. Therefore, AO may be beneficial for facilitating motor recovery for the stroke survivors.

A new interventional strategy, named action observation training (AOT), has been proposed for upper limb motor rehabilitation after stroke [15], which involves action observation and action execution sequentially. In AOT, participants need to observe the actions presented via videos or performed by other people. Afterwards, the participants repetitively stimulate and practice the observed actions [16]. A substantial number of studies regarding the effects of AOT in stroke rehabilitation have been published; however, there is a lack of focused review to quantitatively assess the effects of AOT. The objective of this systematic review and meta-analysis was to assess all available randomized controlled trials to investigate the effects of AOT on upper limb motor functions in people with stroke.

## Materials and methods

### Literature search

In this study, we followed the Preferred Reporting Items for Systematic Review and Meta-Analysis (PRISMA) [17]. Ten databases were selected for this review, including six English-language databases: PubMed, Scopus, EMBASE, PsycINFO (1806+), Cochrane Library, Web of Science; and four Chinese-language databases: China National Knowledge Infrastructure (CNKI), China Biology Medicine (CBM)Database, Wan Fang Database, VIP Database. A systematic literature search was conducted for articles published from January 1^st^, 2000 to March 15^th^, 2019. Articles were identified through comprehensive search of computerized bibliographic databases plus manual searching. The following keywords used exploded Medical Subject Headings (MeSH) terms and text words: (1) “action observation” or “action observation training” or “video therapy”; and (2) “stroke” or “hemiplegia” or “cerebrovascular accident”. The detailed search strategies for each individual electronic database are provided in supplementary file (S2 Table). The reference lists for studies meeting inclusion criteria were also reviewed to identify additional articles for possible inclusion. In order to identify more recent studies that may not be listed in the aforementioned databases, manual searching of the three most recent issues of the journals that had published at least one of the included studies was conducted by BZ (the first author) LK (the coauthor). The search of databases, reference lists and journals occurred between February and March 2019.

### Inclusion and exclusion criteria

Articles were included if they fulfilled all the following criteria: (1) enrolled the clinically diagnosed stroke patients with residual upper limb function impairments, without the limitation in types and stage of stroke; (2) provided multiple sessions of AOT (> 5 sessions) for hemiplegic upper limb motor functions, without the limitation in types of AOT training (observed actions could be presented by video or other people, delivered as either hospital-based training or home-based training); (3) designed as a randomized controlled trial (RCT); (4) study with a control group in which subjects received sham AOT intervention or non-interventional control and (5) published in English or Chinese.

Articles were excluded if they met one of the following criteria: (1) studied healthy subjects or people with neurological diseases excluding stroke; (2) published as a book chapter, degree thesis, review study, study protocol or conference abstract; (3) designed as a single case report or a study without control group and (4) study with insufficient data for meta-analysis.

### Data extraction

Each identified study was first assessed for inclusion and then which was initially summarized by the two authors (BZ and LD) in terms of the following features: (1) characteristics of patients; (2) intervention protocol; (3) primary outcome measures and (4) results of intervention. The accuracy of these summaries was independently checked by another two authors. Disagreements were settled by a discussion with the third author (AD) until these two authors agreed the summary was accurate.

### Methodological quality assessment

The methodological quality of included studies was assessed based on the Risk of Bias Tool in the Cochrane Handbook for Systematic Reviews of Interventions by two independent authors (BZ and LK) [18]: (1) random sequence generation, (2) allocation concealment, (3) blinding of participants and personnel, (4) blinding of outcome assessment, (5) incomplete outcome data, (6) selective reporting, and (7) other bias. Disagreements were resolved by consensus discussion with the third author (AD).

### Meta-analysis

Meta-analysis was conducted using Comprehensive Meta-analysis version 3.0. Authors were contacted by email in case of missing data. Reported standard errors were converted to standard deviations (SD) using the formula SD = SEM × √n (n = sample size). Standardized mean difference (SMD) was computed as the effect size since different tests were used to assess upper limb motor functions across included studies. The effect size was set down as small if the value was between 0.2 and 0.49, medium if the value was between 0.5 and 0.79, and large if the value was more than 0.8 [19]. Using meta-synthesis of post-treatment scores only could result in incorrect findings due to potential baseline difference [20]. Therefore, the change scores (i.e., post-pre) were extracted and used in the estimation of effect size. A random-effects model meta-analysis was performed because of the clinical and methodological heterogeneities across included studies [21]. Subgroup meta-analysis was performed secondarily [22] to explore the effects of different types of AOT (task-based *vs*. movement-based) and the differential effects of AOT in patients with stroke less than 1 month, between 1 to 6 months or over 6 months.

The statistical heterogeneity of studies was assessed by Higgins I^2^ test [23]. There was low heterogeneity if the I^2^ was equal to 25%, moderate heterogeneity if I^2^ was equal to 50%, and high heterogeneity if I^2^ was shown 75% [21]. Publication bias was investigated by visualizing the funnel plots and the Egger’s linear regression test [24], if there were more than 5 studies included in one meta-analysis. Sensitivity analysis was performed by the leave-one-out method [25].

## Results

### Study selection

**Fig 1:**
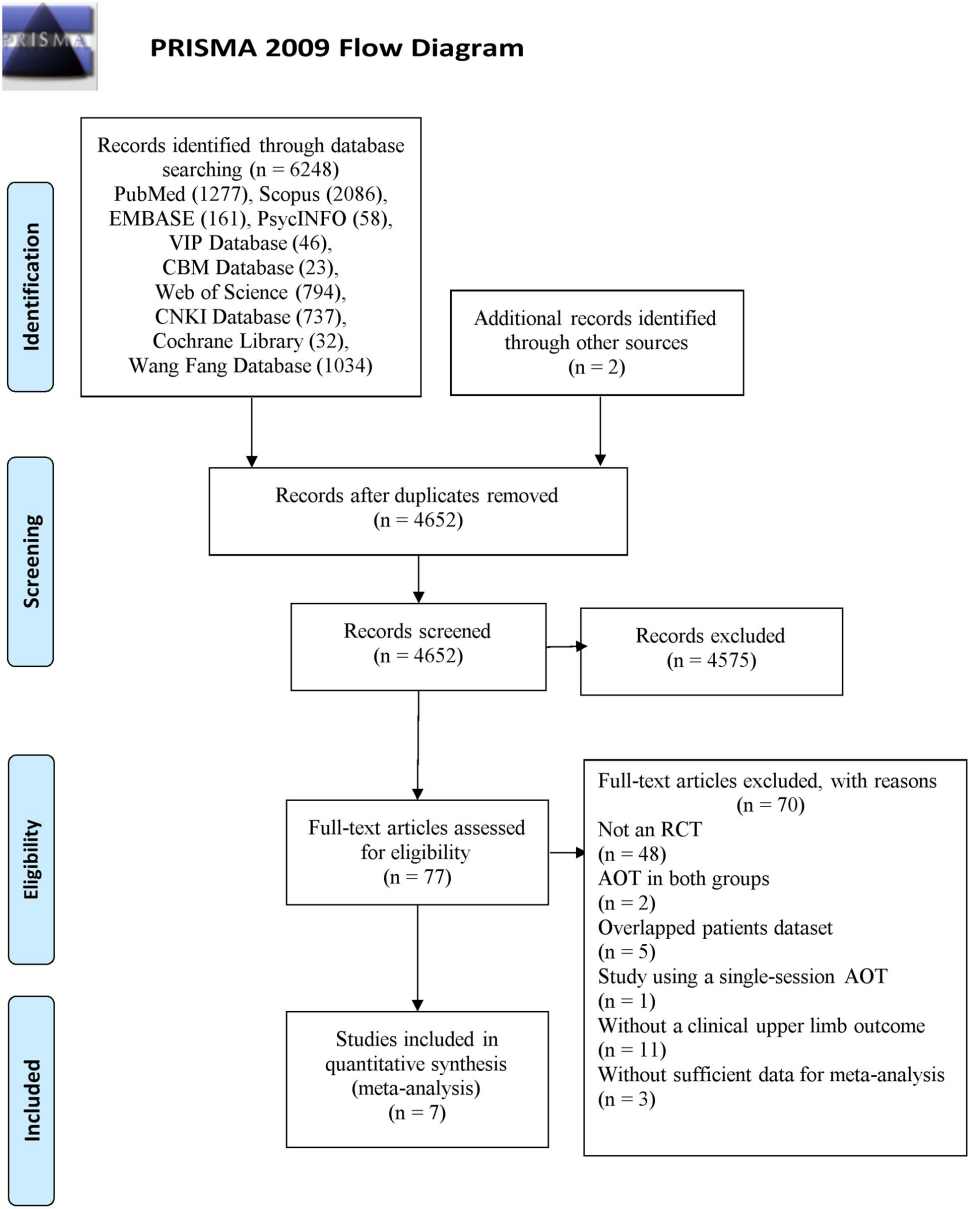
Flow diagram of literature search and recruitment process.

The process of study selection is summarized in Fig 1. Initially, 6250 citations were found in our search, of which, 1598 duplicated citations were excluded. Two independent authors screened the titles and abstracts, and 77 articles were considered as relevant articles. Then, 70 articles were excluded, of which, 48 articles were not RCTs, two articles applied AOT in both groups, one study applied only a single-session AOT, three studies without sufficient data for meta-analysis, five studies reported overlapped patients’ dataset and eleven studies did not use any clinical upper limb motor outcome measurement. Ultimately, seven studies were included in the present review [8, 14, 26–30].

### Characteristics of studies

The characteristics of included studies are summarized in Table 1. Totally, 276 participants with stroke were involved in this review, with 141 participants in the experimental group and 135 participants in the control group. There were two articles with 112 patients who suffered stroke less than 1 month [8, 14], two studies with 75 patients who suffered stroke between 1 and 6 months [27, 30] and one study with 16 patients who suffered stroke over 6 months [26]. One study recruited 61 subjects who suffered stroke within 6 months [29]. Moreover, one study did not elaborate the characteristics of their stroke participants [28].

**Table 1:** Characteristics of included studies.

Study	Group allocation	Duration of stroke	Age (Mean ± SD)	Initial paretic upper limb function	Paretic side (right/left)	Intervention	Intervention duration	Primary outcome (between-group difference)
Ertelt D et al 2007[26]	EG (n = 8); CG (n = 8);	>6 months	EG: 57.16 ± 8.73; CG: 55.40 ± 10.77;	WMFT (time): 2.41 to 41.29 seconds;	4 / 11;	EG: AOT; CG: sham AOT;	90 minutes/session, 1 session/day, 18 days;	FAT: EG = CG (*p* > 0.05);
Franceschini M et al 2012[8]	EG (n = 48); CG (n = 42);	1 month	EG: 67.0 ± 12.4; CG: 65.7 ± 11.9;	Unclear;	50 / 40;	EG: AOT + PT; CG: sham AOT + PT;	15 minutes/session, 2 sessions/day, 5 days/week, 4 weeks;	BBT: EG >CG (*p* < 0.05);
Cowles T et al 2013[14]	EG (n = 9); CG (n = 13);	<1 month	EG: 80.22 ± 9.74; CG: 76.23 ± 12.65;	Grip force: ≤ 65% of healthy side;	7 / 15;	EG: OTI + PP + CPT; CG: CPT;	20 minutes/session, 2 sessions/day, 15 days;	MI: EG = CG (*p* > 0.05);
Zhu M et al 2015[29]	EG (n = 31); CG (n = 30);	<6 months	EG: 57.75 ± 15.57; CG: 56.89 ± 14.93;	FMA ≥ 15;	34 / 27;	EG: AOT + CPT+ OT; CG: CPT+ OT;	30 minutes/session, 6 sessions/week, 8 weeks;	FMA: EG> CG (*p* < 0.05);
Kim E et al 2015[28]	EG (n = 6); CG (n = 6);	Unclear;	Unclear;	Unclear;	Unclear;	EG: AOT + OT; CG: sham AOT + OT;	30 minutes/session, 5 sessions/week, 6 weeks;	WMFT: EG = CG (*p* > 0.05);
Kim C et al 2016[30]	EG (n = 11); CG (n = 11);	1–6 months	EG: 60.77 ± 7.03; CG: 59.11 ± 7.05;	MAS<3	10 / 12	EG: AOT + CRT; CG: TOT+ CRT;	EG: 40 minutes/session, 5 sessions/week, 4 weeks; CG:30 minutes/session, 5 sessions/week, 4 weeks;	FMA: EG>CG (*p* < 0.05);
Fu J et al 2017[27]	EG (n = 28); CG (n = 25);	2–6 months	EG: 62.04 ± 9.93; CG: 59.76 ± 10.57;	FMA ≥ 20;	25 / 28;	EG: CPT + AOT; CG: sham AOT + CPT;	20 minutes/session, 6 sessions/week, 8 weeks;	FMA: EG>CG (*p* < 0.001);

Abbreviations: BBT: Box and block test, WMFT: Wolf Motor Function Test, FMA: Fugl-Meyer assessment, MI: Motricity Index, ARAT: Action Research Arm Test, FAT: Frenchay Arm Test, OT: Occupational therapy, AOT: action observation therapy, CPT: conventional physical therapy, CRT: conventional rehabilitation therapy, OTI: observation-to-imitate, PP: physical practice, PT: physiotherapy, TOT: task-oriented training.

The experimental groups in six studies involved performing task-based AOT, such as grasping a cube, drinking from a glass, combing hair and opening a box [8, 14, 26, 28–30]; while one study conducted movement-based AOT, such as shrugging and adduction of the scapula, bending and extension of the elbow [27]. The control groups in four studies underwent sham AOT, of which, the static landscape paintings were shown to subjects in the control groups [8, 26–28]. Two studies provided the same conventional training for both control and experimental groups, but the latter received AOT in addition [14, 29]. Compared with experimental group, the patients in control group just simulated the predesigned movements without video observation in another study [30]. The duration of the interventions in all studies were ranged from 3 to 8 weeks [8, 14, 26–30], and the majority of them were set as 5 to 6 sessions per week, with each session varying from 20 minutes to 90 minutes.

### Meta-analysis

**Fig 2:**
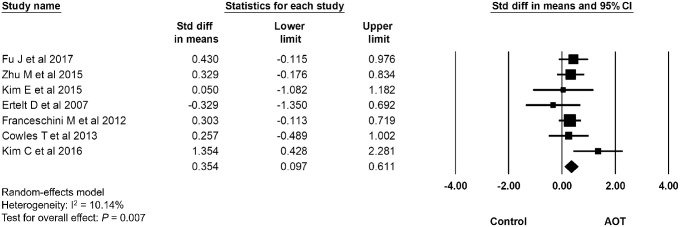
Effects of AOT on upper limb motor functions in patients with stroke.

Seven studies were included in our meta-analysis [8, 14, 26–30]. The result revealed an overall SMD of 0.35 (*p* = 0.007) favoring the AOT over the control, on improving upper limb motor functions in patients with stroke. There was low level of statistical heterogeneity (I^2^ = 10.14%) across trials (Fig 2).

### Subgroup analysis

**Fig 3:**
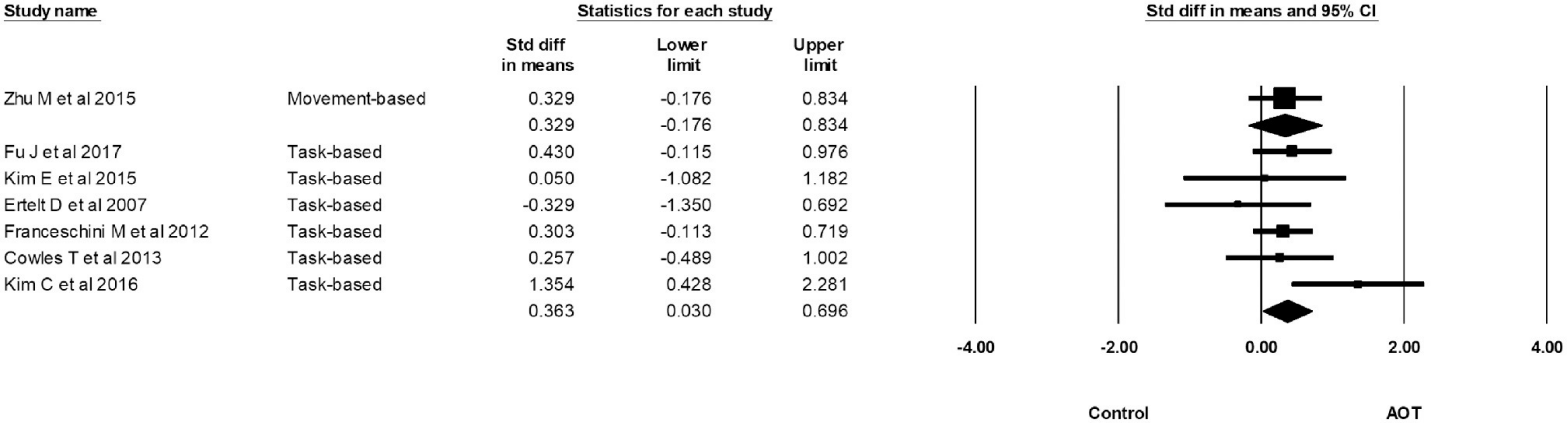
Subgroup analysis of task-based AOT and movement-based AOT.

Subgroup analysis demonstrated that task-based AOT yielded a significant effect on improving upper limb motor functions, with an SMD of 0.36. There was low level of statistical heterogeneity (I^2^ = 25.00%) across trials. Movement-based AOT also yielded an effect size of 0.33; however, its 95% confidence interval [CI] contained zero (Fig 3).

**Fig 4:**
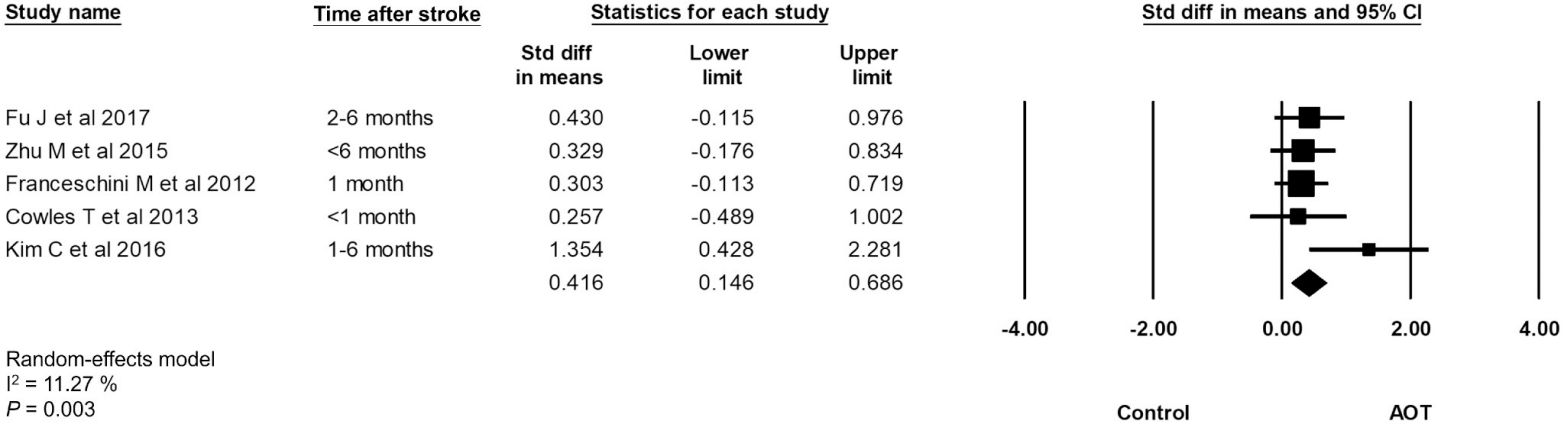
Subgroup analysis of characteristics of stroke.

When considering the characteristics of stroke, subgroup analysis was performed for 5 articles with stroke participants whose course of disease were within 6 months. The results revealed an overall SMD of 0.42 (*p* = 0.003) favoring the AOT was superior to the control in the improvement of upper limb motor functions in stroke patients with course of disease within 6 months (Fig 4).

### Publication bias

**Fig 5:**
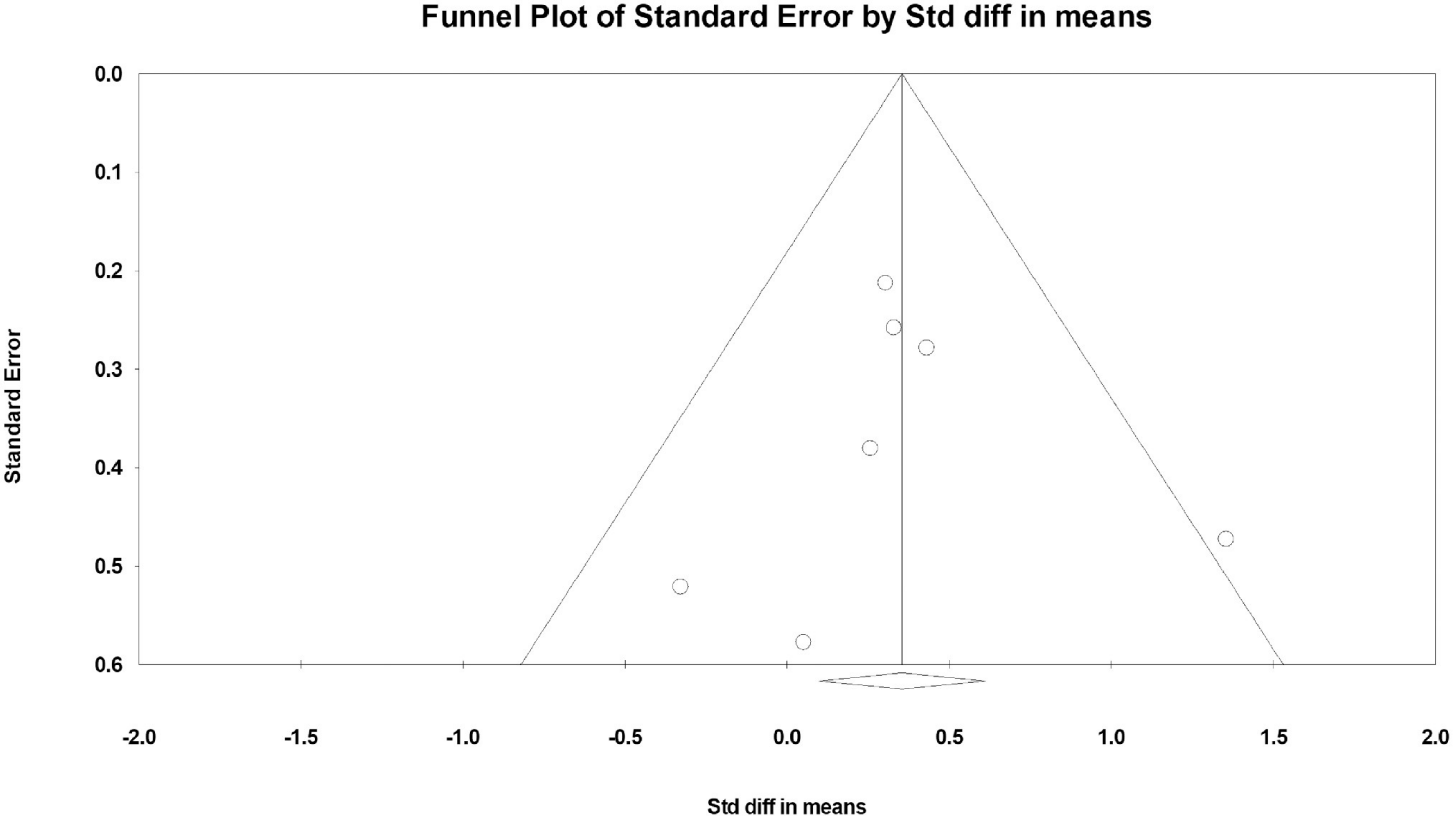
Publication bias.

The funnel plot was generally symmetrical. There was no sign of publication bias (Fig 5), as indicated by the Egger regression test (intercept bias = 0.00, standard error [SE] = 1.30, *p* = 0.99).

### Sensitivity analysis

**Fig 6:**
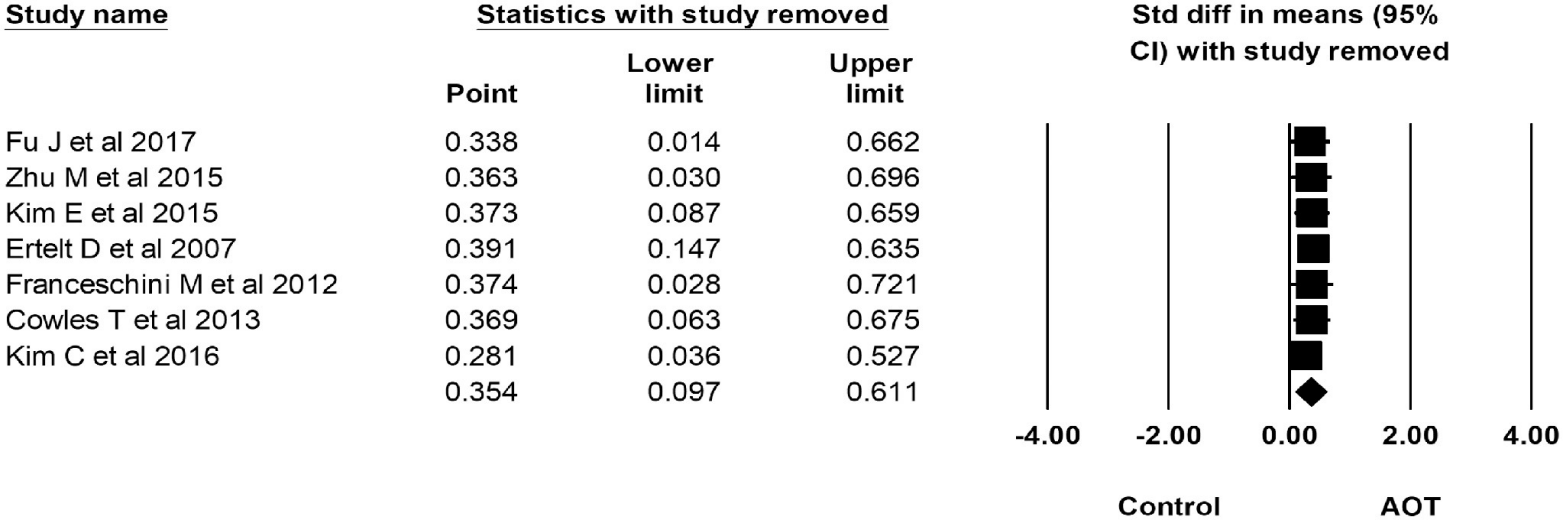
Sensitivity analysis.

The overall effect size remained significant when removing any one of the analyzed trials (Fig 6).

### Meta-regression analysis

Univariate meta-regression analysis was performed when using the total duration of training, number of training sessions and training time per session as the covariates. However, no significant association between above covariates with effect sizes was noted (all *p* > 0.05).

### Methodological quality assessment

**Fig 7:**
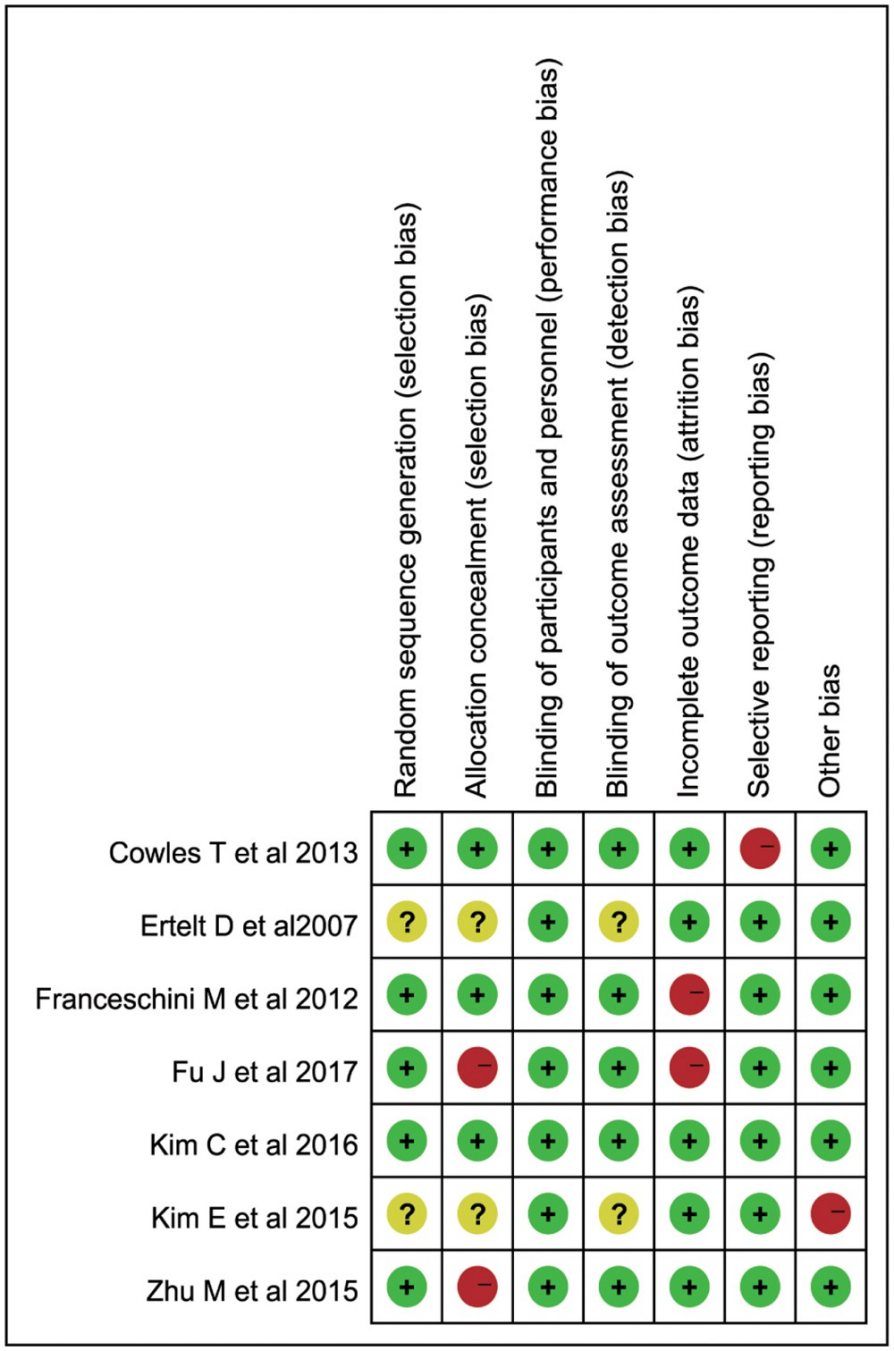
Assessment methodological quality of RCTs.

The methodological quality assessment was summarized in Fig 7. Five studies described the methods of randomization were considered as lower risk of bias [8, 14, 27, 29, 30], while other two studies did not report the details about randomization [26, 28]. Three studies clearly shown the way of allocation concealment [14, 26, 30], while other two studies did not report the information [27, 29]. Due to the nature of AOT, all of the included studies could not blind subjects and therapists. However, it could not have the influence on the treatment effects. Therefore, we consider this criterion in all studies as lower risk of bias. Two studies were considered as high risk of bias in attrition bias because of drop-out cases [8, 26]. One study was considered as high risk of bias in selective reporting [14].

## Discussion

Our meta-analysis showed that AOT had a significant effectiveness on improving the upper limb motor functions immediately after intervention, with a small effect size of 0.35, in patients with stroke. The estimated effect size was robust to leave-one-out sensitivity analysis. However, meta-regression analysis did not find any association between the dosage of AOT and the magnitude of effect sizes.

A previous systematic review published in 2015 has reported that AOT could facilitate motor recovery in patients with neurological diseases [10], but there was a lack of quantitative synthesis. The present review retrieved all RCTs and performed a quantitative analysis on all available data. And we used the primary outcome of each study for meta-analysis, which may optimally reflect the therapeutic effect of the intervention. Although we observed a significant overall effect in favor of AOT, the results of meta-regression showed no evidence of cumulative effect of AOT. The possible reasons include: (1) The majority of studies were conducted in patients who suffered stroke within 6 months, the spontaneous recovery and motor recovery benefited from customary training may confound the effects of AOT; (2) The improvement curve associating with AOT may not be in a linear manner. The rate of improvement may be more prominent in the first few sessions but becomes limited due to adaptations; (3) The number of studies (n = 7) was not enough for a linear regression analysis.

As the absence of adequate follow-up data among identified studies, our review just examined the short-term effects of AOT in meta-analysis. Among our included studies, only one study investigated the long-term effects of AOT [8] and it reported that patients in AOT group demonstrated a significantly higher upper-limb functional score in Box and Block Test than control group, while the effects maintained in follow-up [8]. This finding indicates the substantiality of effects of AOT on improving upper limb motor functions. Further studies are encouraged to perform follow-up session in order to determine the substantiality of effects of AOT.

The majority of studies employed task-based AOT except one, which used movement-based AOT. Task-based AOT may be more effective, in terms of activating the mirror neurons, since mirror neurons are more receptive to object-related actions [31, 32]. Task-based AOT and movement-based AOT yielded a similar effect size according to the results of this meta-analysis. Yet, movement-based AOT was used in only one clinical trial [29] and the results remained insignificant. Further studies should directly compare the effects of task-based AOT and movement-based AOT in order to determine the superiority.

The neural mechanism underlying the AOT was rarely investigated. Ertelt et al. [26] were the first who investigated the brain activation accompanying with the AOT. A higher activation in the supplementary motor area, bilateral ventral premotor cortex, bilateral superior and inferior parietal areas was found after AOT, which supported the mirror neurons hypothesis. The relationship between mirror neurons activation and upper limb motor recovery in stroke survivors has been revealed in a longitudinal neuroimaging study [33], although the evidence is still too limited. The activation of the ipsilesional primary motor cortex has been shown to be associated with the upper limb motor recovery among stroke survivors by a large body of neuroimaging studies [34]. Fu et al. reported the increment of motor evoked potentials (MEP) collected from hemiplegic upper limbs in patients with stroke that received the AOT [27], which supported the effects of AOT was likely to be attributed to the activation of the ipsilesional primary motor cortex. Taken together, AOT may facilitate the upper limb motor recovery by activating the mirror neurons, which makes the ipsilesional motor cortex more receptive to the motor training. More robust neuroimaging studies are needed to confirm the speculation.

We need to interpret the results cautiously due to the potential bias of included studies. All involved studies could not blind subjects and therapists because of the way of conducting AOT. There was a methodological limitation related to sham AOT, which was declared in four included studies [8, 26–28]. Patients who were receiving sham AOT were likely to recognize that they did not receive real interventions, which may contribute to the overestimation of the effects of AOT.

### Limitations

Our study is not free from limitations. Firstly, the number of included studies was limited. The results need to be replicated in further dedicated studies. Secondly, although we extracted the primary outcomes of each study to avoid the selection bias of investigators, used the change score to avoid potential bias caused by baseline difference, and standardized the effect size in meta-analysis, the estimation of effect sizes still may be confounded because different measurements were used. Thirdly, the participants varied in age and other characteristics, which may bias their response to intervention. However, the effects of these confounding factors on treatment effects remained unknown. Lastly, although we examined the publication bias by funnel plot and Egger test, we only limited our search to the literature in either English or Chinese language. Therefore, the review may be at the risk of language bias.

## Conclusion

AOT is an effective method for improving upper limb motor function after stroke, and task-based AOT might be superior to movement-based AOT. The optimal dosage, the substantiality of effects and the underlying neural mechanisms of AOT on improving upper limb motor functions in patients with stroke should be considered in further investigation.

## Supporting information

S1 Table Checklist. PRISMA 2009 checklist.
(DOC)

S2 Table Search strategies for each electronic database. (DOCX)

S3 Table Minimal data set of all included studies. (DOCX)
