# Supplementary material for: The effects of action observation training on improving upper limb motor functions in people with stroke: A systematic review and meta-analysis
Source: PLoS One. 2019 Aug 30;14(8):e0221166. doi: 10.1371/journal.pone.0221166 (PMC6716645; doi:10.1371/journal.pone.0221166)
Supplement: S2 Table — (DOCX) [file pone.0221166.s002.docx]

Manuscript submitted to ***PLoS ONE***

**The effects of action observation training on improving upper limb motor functions in people with stroke: A systematic review and meta-analysis**

Bingbing Zhang^1,2¶^, Laidi Kan^1¶^, Anqin Dong^1*,#a^, Jiaqi Zhang^3^, Zhongfei Bai^3,4^, Yi Xie ^5^, Qianhao Liu^1^ and Yuzhong Peng^1^

Supplementary sections

| Table S1. PubMed Search Strategy. | Page 2 |
| --- | --- |
| Table S2. EMBASE Search Strategy. | Page 3 |
| Table S3. Cochrane Library Search Strategy. | Page 4 |
| Table S4. Scopus Search Strategy. | Page 5 |
| Table S5. PsycINFO (1806+) Search Strategy. | Page 6 |
| Table S6. Web of Science Search Strategy. | Page 7 |
| Table S7. CNKI Database Search Strategy. | Page 8 |
| Table S8. CBM Database Search Strategy. | Page 9-10 |
| Table S9. Wang Fang Database Search Strategy. | Page 11 |
| Table S10. VIP Database Search Strategy. | Page 12 |

**Table S1. PubMed Search Strategy.**

| **Step** | **Search strategy** | **Results** |
| --- | --- | --- |
| #7 | (((((cerebrovascular accident AND ("2000/01/01" [PDat]: ''3000/12/31' [PDat]))) OR (((("hemiplegia" [Mesh] AND ("2000/01/01" [PDat]: ''3000/12/31' [PDat]))) OR ("stroke" [Mesh] AND ("2000/01/01" [PDat]: ''3000/12/31' [PDat]))) AND ("2000/01/01" [PDat]: ''3000/12/31' [PDat]))) AND ("2000/01/01" [PDat]: ''3000/12/31' [PDat]))) AND (((( action observation training) OR action observation) OR video therapy) AND ("2000/01/01" [PDat]: ''3000/12/31' [PDat])) Filters: Publication date from 2000/01/01 | 1277 |
| #6 | ((cerebrovascular accident AND ("2000/01/01" [PDat]: ''3000/12/31' [PDat])) OR (((("hemiplegia" [Mesh] AND ("2000/01/01" [PDat]: ''3000/12/31' [PDat]))) OR ("stroke" [Mesh] AND ("2000/01/01" [PDat]: ''3000/12/31' [PDat]))) AND ("2000/01/01" [PDat]: ''3000/12/31' [PDat])) Filters: Publication date from 2000/01/01 | 241986 |
| #5 | cerebrovascular accident Filters: Publication date from 2000/01/01 | 240340 |
| #4 | (("hemiplegia" [Mesh] AND ("2000/01/01" [PDat]: ''3000/12/31' [PDat]))) OR ("stroke" [Mesh] AND ("2000/01/01" [PDat]: ''3000/12/31' [PDat])) Filters: Publication date from 2000/01/01 | 107537 |
| #3 | "hemiplegia" [Mesh] Filters: Publication date from 2000/01/01 | 3244 |
| #2 | "stroke" [Mesh] Filters: Publication data from 2000/01/01 | 105587 |
| #1 | ((action observation training) OR action observation) OR video therapy Filters: Publication date from 2000/01/01 | 47459 |

**Table S2. EMBASE Search Strategy.**

| **Step** | **Search strategy** | **Results** |
| --- | --- | --- |
| #7 | #1 AND #6 [2000-2019]/py | 161 |
| #6 | #2 OR #5 | 463659 |
| #5 | #3 OR #4 | 313989 |
| #4 | 'cerebrovascular accident' / exp | 299667 |
| #3 | 'hemiplegia' / exp | 19001 |
| #2 | 'stroke 'AND [2000-2019]/py | 356386 |
| #1 | ('action observation training' OR 'action observation' OR 'video therapy') AND [2000-2019]/py | 1379 |

**Table S3. Cochrane Library Search Strategy.**

| **Step** | **Search strategy** | **Results** |
| --- | --- | --- |
| #7 | #1 AND #6 (with Cochrane Library publication date from Jan 2000 to Mar 2019) | 32 |
| #6 | #4 OR #5 | 16472 |
| #5 | ("cerebrovascular accident") | 9096 |
| #4 | #2 OR #3 | 8382 |
| #3 | MeSH descriptor: [hemiplegia] explode all trees | 631 |
| #2 | MeSH descriptor: [stroke] explode all trees | 8034 |
| #1 | ("action observation training") OR ("action observation") OR ("video therapy") | 164 |

**Table S4. Scopus Search Strategy.**

| **Step** | **Search Term(s)** | **Results** |
| --- | --- | --- |
| #3 | #1 AND #2 | 2086 |
| #2 | (ALL (stroke) OR ALL (hemiplegia) OR ALL ("cerebrovascular accident")) AND PUBYEAR > 1999 | 951664 |
| #1 | (ALL ("action observation") OR ALL ("action observation training") OR ALL ("video therapy") AND PUBYEAR >1999 | 8724 |

**Table S5. PsycINFO (1806+) Search Strategy.**

| **Step** | **Search strategy** | **Results** |
| --- | --- | --- |
| S5 | S1 AND S4 | 58 |
| S4 | S2 OR S3 | 52035 |
| S3 | stroke | 50107 |
| S2 | MAINSUBJECT. EXACT.EXPLODE ("cerebrovascular accidents") OR MAINSUBJECT. EXACT. EXPLODE ("hemiplegia") | 20372 |
| S1 | "action observation training" OR "action observation" OR "video therapy" | 965 |

*Publication time was limited to between 1^st^ February 2000 and 15^th^ March 2019.*

**Table S6. Web of Science Search Strategy.**

| **Step** | **Search Term(s)** | **Results** |
| --- | --- | --- |
| #3 | #1 *AND* #2  *Indexes = SCI-EXPANDED, SSCI, A&HCI, CPCI-S, CPCI-SSH, ESCI Timespan = 2000-2019* | 794 |
| #2 | (ALL FIELDS: (stroke) *OR* ALL FIELDS: (hemiplegia) *OR* ALL FIELDS: (cerebrovascular accident))  *Indexes = SCI-EXPANDED, SSCI, A&HCI, CPCI-S, CPCI-SSH, ESCI Timespan = 2000-2019* | 278258 |
| #1 | (ALL FIELDS: (action observation) *OR* ALL FIELDS: (action observation training) *OR* ALL FIELDS: (video therapy)  *Indexes = SCI-EXPANDED, SSCI, A&HCI, CPCI-S, CPCI-SSH, ESCI Timespan = 2000-2019* | 34069 |

**Table S7. CNKI Database Search Strategy.**

| [发表时间 between (2000-01-01,2019-03-15) 并且 ( ( ( ( ( 主题=同义词扩展(卒中) 或者 主题=同义词扩展(中风) ) 或者 ( 题名=同义词扩展(卒中) 或者 题名=同义词扩展(中风) ) ) 或者 ( ( 主题=同义词扩展(脑梗死) 或者 主题=同义词扩展(脑缺血) ) 或者 ( 题名=同义词扩展(脑梗死) 或者 题名=同义词扩展(脑缺血) ) ) ) 或者 ( ( 主题=同义词扩展(脑出血) 或者 主题=同义词扩展(偏瘫) ) 或者 ( 题名=同义词扩展(脑出血) 或者 题名=同义词扩展(偏瘫) ) ) ) 或者 ( ( 主题=同义词扩展(脑血管意外) 或者 主题=同义词扩展(脑血管障碍) ) 或者 ( 题名=同义词扩展(脑血管意外) 或者 题名=同义词扩展(脑血管障碍) ) ) ) 并且 发表时间 between (2000-01-01,2019-03-15) 并且 ( ( ( ( 主题=同义词扩展(动作观察) 或者 主题=同义词扩展(动作观察疗法) ) 或者 ( 题名=同义词扩展(动作观察) 或者 题名=同义词扩展(动作观察疗法) ) ) 或者 ( ( 主题=同义词扩展(运动观察) 或者 主题=同义词扩展(运动观察疗法) ) 或者 ( 题名=同义词扩展(运动观察) 或者 题名=同义词扩展(运动观察疗法) ) ) ) 或者 (主题=同义词扩展(视频训练) 或者 题名=同义词扩展(视频训练)) ) (模糊匹配),专辑导航：全部; 数据库：文献 跨库检索](http://new.gb.oversea.cnki.net.ezproxy.lb.polyu.edu.hk/kns/brief/ShowHistorySelect.aspx?QueryID=9&dbCatalog=%e6%96%87%e7%8c%ae&DBPREFIX=SCDB&condition=%e6%a3%80%e7%b4%a2%e6%9d%a1%e4%bb%b6%ef%bc%9a%e5%8f%91%e8%a1%a8%e6%97%b6%e9%97%b4+between+(2000-01-01%2c2019-03-15)+++%e5%b9%b6%e4%b8%94+++(+(+(+(+(+%e4%b8%bb%e9%a2%98%3d%e5%90%8c%e4%b9%89%e8%af%8d%e6%89%a9%e5%b1%95(%e5%8d%92%e4%b8%ad)++++%e6%88%96%e8%80%85++++%e4%b8%bb%e9%a2%98%3d%e5%90%8c%e4%b9%89%e8%af%8d%e6%89%a9%e5%b1%95(%e4%b8%ad%e9%a3%8e)+)++++%e6%88%96%e8%80%85++++(+%e9%a2%98%e5%90%8d%3d%e5%90%8c%e4%b9%89%e8%af%8d%e6%89%a9%e5%b1%95(%e5%8d%92%e4%b8%ad)++++%e6%88%96%e8%80%85++++%e9%a2%98%e5%90%8d%3d%e5%90%8c%e4%b9%89%e8%af%8d%e6%89%a9%e5%b1%95(%e4%b8%ad%e9%a3%8e)+)+)+++%e6%88%96%e8%80%85++++(+(+%e4%b8%bb%e9%a2%98%3d%e5%90%8c%e4%b9%89%e8%af%8d%e6%89%a9%e5%b1%95(%e8%84%91%e6%a2%97%e6%ad%bb)++++%e6%88%96%e8%80%85++++%e4%b8%bb%e9%a2%98%3d%e5%90%8c%e4%b9%89%e8%af%8d%e6%89%a9%e5%b1%95(%e8%84%91%e7%bc%ba%e8%a1%80)+)++++%e6%88%96%e8%80%85++++(+%e9%a2%98%e5%90%8d%3d%e5%90%8c%e4%b9%89%e8%af%8d%e6%89%a9%e5%b1%95(%e8%84%91%e6%a2%97%e6%ad%bb)++++%e6%88%96%e8%80%85++++%e9%a2%98%e5%90%8d%3d%e5%90%8c%e4%b9%89%e8%af%8d%e6%89%a9%e5%b1%95(%e8%84%91%e7%bc%ba%e8%a1%80)+)+)++)+++%e6%88%96%e8%80%85++++(+(+%e4%b8%bb%e9%a2%98%3d%e5%90%8c%e4%b9%89%e8%af%8d%e6%89%a9%e5%b1%95(%e8%84%91%e5%87%ba%e8%a1%80)++++%e6%88%96%e8%80%85++++%e4%b8%bb%e9%a2%98%3d%e5%90%8c%e4%b9%89%e8%af%8d%e6%89%a9%e5%b1%95(%e5%81%8f%e7%98%ab)+)++++%e6%88%96%e8%80%85++++(+%e9%a2%98%e5%90%8d%3d%e5%90%8c%e4%b9%89%e8%af%8d%e6%89%a9%e5%b1%95(%e8%84%91%e5%87%ba%e8%a1%80)++++%e6%88%96%e8%80%85++++%e9%a2%98%e5%90%8d%3d%e5%90%8c%e4%b9%89%e8%af%8d%e6%89%a9%e5%b1%95(%e5%81%8f%e7%98%ab)+)+)++)+++%e6%88%96%e8%80%85++++(+(+%e4%b8%bb%e9%a2%98%3d%e5%90%8c%e4%b9%89%e8%af%8d%e6%89%a9%e5%b1%95(%e8%84%91%e8%a1%80%e7%ae%a1%e6%84%8f%e5%a4%96)++++%e6%88%96%e8%80%85++++%e4%b8%bb%e9%a2%98%3d%e5%90%8c%e4%b9%89%e8%af%8d%e6%89%a9%e5%b1%95(%e8%84%91%e8%a1%80%e7%ae%a1%e9%9a%9c%e7%a2%8d)+)++++%e6%88%96%e8%80%85++++(+%e9%a2%98%e5%90%8d%3d%e5%90%8c%e4%b9%89%e8%af%8d%e6%89%a9%e5%b1%252) | 737 |
| --- | --- |

**Table S8. CBM Database Search Strategy.**

| **Step** | **Search strategy** | **Results** |
| --- | --- | --- |
| #17 | (#6) AND (#16) | 23 |
| #16 | (#7) OR (#14) | 417296 |
| #15 | (#8) OR (#9) OR (#10) OR (#11) OR (#12) OR (#13) OR (#14) | 401842 |
| #14 | "脑血管障碍"[不加权：扩展] | 381343 |
| #13 | "偏瘫"[不加权：扩展] | 21132 |
| #12 | "脑出血"[不加权：扩展] | 53742 |
| #11 | "脑缺血"[不加权：扩展] | 171762 |
| #10 | "脑梗死"[不加权：扩展] | 98892 |
| #9 | "中风"[不加权：扩展] | 17641 |
| #8 | "卒中"[不加权：扩展] | 207788 |
| #7 | ''脑血管意外'' [全字段：智能] | 233987 |
| #6 | (#1) OR (#2) OR (#3) OR (#4) OR (#5) | 72 |
| #5 | ''视频训练'' [全字段：智能] | 6 |
| #4 | ''运动观察疗法'' [全字段：智能] | 3 |
| #3 | ''运动观察'' [全字段：智能] | 39 |
| #2 | ''动作观察疗法'' [全字段：智能] | 12 |
| #1 | ''动作观察'' [全字段：智能] | 28 |

**Table S9. Wang Fang Database Search Strategy.**

| (题名或关键词:(卒中 OR 中风 OR 脑出血 OR 脑梗死 OR 脑缺血 OR 偏瘫 OR 脑血管意外 OR 脑血管障碍)*题名或关键词:(运动观察疗法 OR 运动观察 OR 动作观察疗法 OR 动作观察 OR 视频训练))*Date:2000-2019 | 1034 |
| --- | --- |

**Table S10. VIP Database Search Strategy.**

| [((((((((题名或关键词=卒中 OR 题名或关键词=中风) OR 题名或关键词=脑出血) OR 题名或关键词=脑梗死) OR 题名或关键词=脑缺血) OR 题名或关键词=偏瘫) OR 题名或关键词=脑血管意外) OR 题名或关键词=脑血管障碍) AND ((((题名或关键词=运动观察疗法 OR 题名或关键词=运动观察) OR 题名或关键词=动作观察疗法) OR 题名或关键词=动作观察) OR 题名或关键词=视频训练)) AND (years:[2000 TO 2019])](https://vpn2.zzu.edu.cn/Qikan/search/,DanaInfo=qikan.cqvip.com+index?LngMySearHistoryIdGuid=3d9fc58d-2aef-4cdb-bb5f-79598ef8143c&from=Qikan_Article_History) | 46 |
| --- | --- |
