# Supplementary material for: The effects of action observation training on improving upper limb motor functions in people with stroke: A systematic review and meta-analysis
Source: PLoS One. 2019 Aug 30;14(8):e0221166. doi: 10.1371/journal.pone.0221166 (PMC6716645; doi:10.1371/journal.pone.0221166)
Supplement: S3 Table — (DOCX) [file pone.0221166.s003.docx]

Manuscript submitted to ***PLoS ONE***

**The effects of action observation training on improving upper limb motor functions in people with stroke: A systematic review and meta-analysis**

Bingbing Zhang^1,2¶^, Laidi Kan^1¶^, Anqin Dong^1*,#a^, Jiaqi Zhang^3^, Zhongfei Bai^3,4^, Yi Xie ^5^, Qianhao Liu^1^ and Yuzhong Peng^1^

Supplementary sections

| Table S11. Minimal data set of all included studies. | Page 2 |
| --- | --- |

**Table S11. Minimal data set of all included studies.**

| **Study** | **Outcome** | **Pre-assessment (Mean ± SD)** | **Post-assessment (Mean ± SD)** |
| --- | --- | --- | --- |
| Ertelt D et al 2007 | FAT | EG = 10.88 ± 8.30;  CG = 16.67 ± 14.99 | EG = 7.04 ± 6.86;  CG = 16.97 ± 15.94 |
| Franceschini M et al 2012 | BBT | EG = 8.50 ± 12.20;  CG = 8.30 ± 10.50 | EG = 20.00 ± 19.20;  CG = 14.50 ± 15.30 |
| Cowles T et al 2013 | MI | Change EG = 9.54 ± 8.98;  Change CG = 7.07 ± 10.15 | / |
| Zhu M et al 2015 | FMA | EG = 27.58 ± 9.53;  CG = 28.77 ± 9.80 | EG = 35.52 ± 12.43;  CG = 32.80 ± 11.29 |
| Kim E et al 2015 | WMFT | EG = 32.80 ± 10.80;  CG = 35.60 ± 15.10 | EG = 39.80 ± 12.20;  CG = 42.00 ± 11.67 |
| Kim C et al 2016 | FMA | EG = 40.00 ± 2.59;  CG = 39.89 ± 3.66 | EG = 52.78 ± 3.35;  CG = 48.44 ± 2.88 |
| Fu J et al 2017 | FMA | EG = 31.46 ± 10.66;  CG = 29.60 ± 12.29 | EG = 42.32 ± 12.56;  CG = 35.08 ± 12.44 |

Abbreviations: BBT: Box and block test, WMFT: Wolf Motor Function Test, FMA: Fugl-Meyer assessment, MI: Motricity Index, ARAT: Action Research Arm Test, FAT: Frenchay Arm Test.
